# Facile Fabrication of Superhydrophobic Cellulose Film with Enhanced Abrasion and Corrosion Resistance for High-Flux Oil-Water Separation

**DOI:** 10.3390/molecules31203678

**Published:** 2026-10-14

**Authors:** Rongjie Huang, Xinchu Lu, Honghuan Gan, Tulian Li, Zaiying Zheng, Yanzhen Yin, Daguang He

**Affiliations:** 1 College of Petroleum and Chemical Engineering, Beibu Gulf University, Qinzhou 515000, China; 2 Guangxi Key Laboratory of Green Chemical Materials and Safety Technology, Beibu Gulf University, Qinzhou 515000, China

**Keywords:** cellulose, polydopamine, long-chain silane, superhydrophobic materials

## Abstract

A durable and environmentally friendly superhydrophobic cellulose-based membrane, denoted as FP/PDA@MCC/OTS (where FP, PDA, MCC, and OTS represent filter paper, polydopamine, microcrystalline cellulose, and octadecyltrichlorosilane, respectively), was fabricated via a simple PDA toughening and OTS-modified MCC coating strategy. Cellulose filter paper was first reinforced with a PDA interfacial layer to enhance mechanical stability, adhesion, and chemical resistance. Subsequently, hydrophobic MCC particles grafted with OTS were spray-deposited, forming a hierarchical fiber-particle composite structure. The resulting membrane exhibits a stable superhydrophobic surface with a water contact angle (WCA) above 150°, attributed to the synergistic effect of hierarchical roughness and low-surface-energy modification. It achieves high oil-water separation efficiency (>98%, up to 99.99%) and a high flux of up to 2.3 × 10^4^ L·m^−2^·h^−1^ under gravity-driven conditions. Moreover, the membrane maintains excellent performance stability after repeated abrasion, ultrasonic treatment, and long-term acid/alkali immersion, with WCA remaining above 136°. The enhanced durability is attributed to robust interfacial interactions provided by PDA and stable surface anchoring of OTS-derived silane species on cellulose. This work provides a simple, scalable, and sustainable strategy for constructing robust cellulose-based membranes for efficient oily wastewater treatment under harsh conditions.

## 1. Introduction

The escalating discharge of oily wastewater from industrial processes, coupled with frequent offshore oil spill accidents, has imposed severe threats to aquatic ecosystems and public health [1,2]. Developing efficient, cost-effective, and environmentally sustainable oil-water separation technologies has therefore become an urgent global priority. Among the available approaches, membrane-based separation stands out due to its high efficiency, low energy consumption, operational simplicity, and scalability, particularly for treating emulsified oil-water systems [3,4,5]. The separation performance of such membranes is largely governed by surface wettability and interfacial microstructure. By engineering surface chemistry and micro/nanoscale topography, membranes can be designed to exhibit either superhydrophilic/underwater superoleophobic or superhydrophobic/superoleophilic properties, enabling selective permeation of water or oil.

Despite significant progress, many conventional superhydrophilic membranes—such as catechol/chitosan-modified polymers and fluorinated covalent organic framework (COF) coatings-face inherent limitations including poor biodegradability, environmental persistence, and potential secondary pollution associated with synthetic polymers and fluorinated compounds [6,7,8,9,10]. These concerns have motivated a paradigm shift toward sustainable, bio-derived membrane materials. Cellulose, the most abundant natural polymer on Earth, has attracted particular interest owing to its renewability, biodegradability, low cost, and abundant surface hydroxyl groups that facilitate hydrogen bonding and chemical functionalization [11]. These features make cellulose an ideal platform for constructing filtration membranes with tunable wettability and robust mechanical properties [12,13,14]. Among the several types of cellulose, microcrystalline cellulose (MCC) is especially advantageous as a functional particle for spray-coating due to its insolubility in water and typical organic solvents (ethanol, hexane), enabling the formation of stable suspensions without dissolution or gelation. Conversely, soluble celluloses (such as CMC-Na, methylcellulose, HPMC) tend to swell or dissolve when exposed to aqueous environments, hence undermining the structural stability of the coating during oil-water separation. The 8–10 μm particle dimension and plentiful surface hydroxyl groups of MCC yield a significant specific surface area and reactive sites for covalent silane grafting, rendering it an optimal component for developing hierarchical hydrophobic coatings.

Considerable efforts have been devoted to developing cellulose-based oil-water separation membranes. Zhang et al. [15] fabricated a lignin-reinforced cellulose filter paper capable of simultaneous oil-in-water emulsion separation and dye removal via aldol condensation and dopamine cross-linking. Cheng et al. [16] designed superhydrophilic polylactic acid (PLA) nanofiber membranes integrated with polyethylene oxide hydrogels, achieving a 61.9-fold increase in emulsion permeance. Gao et al. [17] utilized pulp refining and in situ gelation to prepare all-cellulose paper membranes with >98.5% separation efficiency. Chen et al. [18] reported a superhydrophobic rice starch/shellac composite coating with a water contact angle (WCA) of 153.1°. Despite these advances, pristine cellulose remains intrinsically hydrophilic, necessitating rational surface modification to achieve selective oil/water wettability.

To address this challenge, various modification strategies have been explored. Tunicate cellulose nanocrystal-modified filter papers have demonstrated superhydrophilic/underwater superoleophobic properties with >99% emulsion separation efficiency [19]. Polydopamine (PDA)-assisted interfacial engineering has emerged as a versatile platform for depositing functional nanoparticles (e.g., Ag) and subsequent surface functionalization, offering additional antibacterial and catalytic functionalities [20,21]. Various bio-derived coatings, such as those based on alginate, hydroxyapatite, natamycin, and beeswax, have also been developed [22]. However, a common bottleneck persists across many of these systems: insufficient mechanical durability and interfacial adhesion stability. Under practical operating conditions, long-term abrasion, ultrasonic cavitation, and acid/alkali corrosion inevitably lead to coating delamination and performance degradation [23]. This durability limitation stems from an inherent wetting issue: unaltered cellulose filter paper is naturally hydrophilic (WCA ≈ 0°) and swiftly absorbs water through capillary action, resulting in fiber expansion and pore obstruction; oil is unable to penetrate the water-saturated pores, resulting in membrane malfunction. Consequently, the objective of superhydrophobic alteration is not solely to reject water, but to create a “selective wetting window”—inhibiting water penetration while permitting oil (surface tension ≈ 30 mN/m compared to 72 mN/m for water) to pass unobstructed through the hydrophobic pores. Among organosilane modifiers, octadecyltrichlorosilane (OTS) was selected over shorter-chain silanes (such as TMCS and APTES) due to its C18 alkyl chain, which offers the lowest surface energy (approximately 20 mN/m) for achieving superhydrophobicity (greater than 150°), while its trichloro functionality enables multipoint surface anchoring onto cellulose hydroxyl groups via possible Si-O-C linkages and Si-O-Si cross-linked siloxane networks, contributing to improved chemical stability under harsh acidic or alkaline environments (pH 1–14).

Most recently, Ning et al. [23] reported a superhydrophobic cellulose paper prepared via a two-step immersion method using octadecyltrichlorosilane (OTS) and hydrophobic SiO_2_ nanoparticles, achieving a WCA of 157° and a separation efficiency of 99.31% with a flux of ≈6100 L·m^−2^·h^−1^. Although this represented a significant advance, their coating suffered from gradual degradation after only 50 abrasion cycles, highlighting the critical need for stronger interfacial binding between hydrophobic coatings and cellulose substrates. This limitation motivated our present design: can we introduce a tough PDA interlayer to reinforce particle-substrate adhesion while maintaining the superhydrophobic function?

We present a simple and scalable method to create a resilient superhydrophobic cellulose membrane (FP/PDA@MCC/OTS) by overcoming the primary drawback of earlier techniques—the absence of a strong interfacial adhesive layer that secures hydrophobic particles to flexible cellulose substrates during mechanical stress. Our first assumption is that the incorporation of a polydopamine (PDA) interlayer between the cellulose paper and the OTS-grafted MCC coating will markedly improve particle-substrate adhesion and the overall resilience of the membrane, while maintaining its superhydrophobic properties.

In this configuration, microcrystalline cellulose (MCC) powder is chemically modified with OTS to produce hydrophobic MCC/OTS particles, while a polydopamine (PDA) layer is initially applied to cellulose filter paper (FP) to improve mechanical integrity and interfacial bonding. Thereafter, MCC/OTS particles are applied via spray coating onto the PDA-altered substrate, resulting in a composite structure characterized by hierarchical fiber and particle integration.

This integrated design offers four synergistic benefits: (i) the hierarchical roughness from the fiber-particle assembly confers superhydrophobicity with a WCA exceeding 150°; (ii) the low-surface-energy OTS grafting guarantees elevated oil selectivity; (iii) the PDA interlayer ensures robust interfacial interactions (hydrogen-bonding and catechol-mediated adhesion) that effectively resists coating detachment under mechanical stress; and (iv) the resultant membrane demonstrates high separation efficiency (over 98%, reaching 99.99%) and substantial flux (up to 2.3 × 10^4^ L·m^−2^·h^−1^) under gravity-driven conditions, while exhibiting remarkable stability after repeated abrasion, ultrasonic treatment, and prolonged acid/alkali immersion.

In contrast to the recently documented OTS-based cellulose systems that experience swift performance decline after merely 40–50 abrasion cycles under 5–10 g loads, our PDA-reinforced approach is anticipated to provide enhanced mechanical resilience under greater loads while preserving similar or even elevated separation flux. This study offers an uncomplicated, scalable, and sustainable method for fabricating durable cellulose-based membranes appropriate for effective oily wastewater treatment in challenging environments.

## 2. Results and Discussion

### 2.1. FTIR, XRD and XPS Characterization

**Figure 1:**
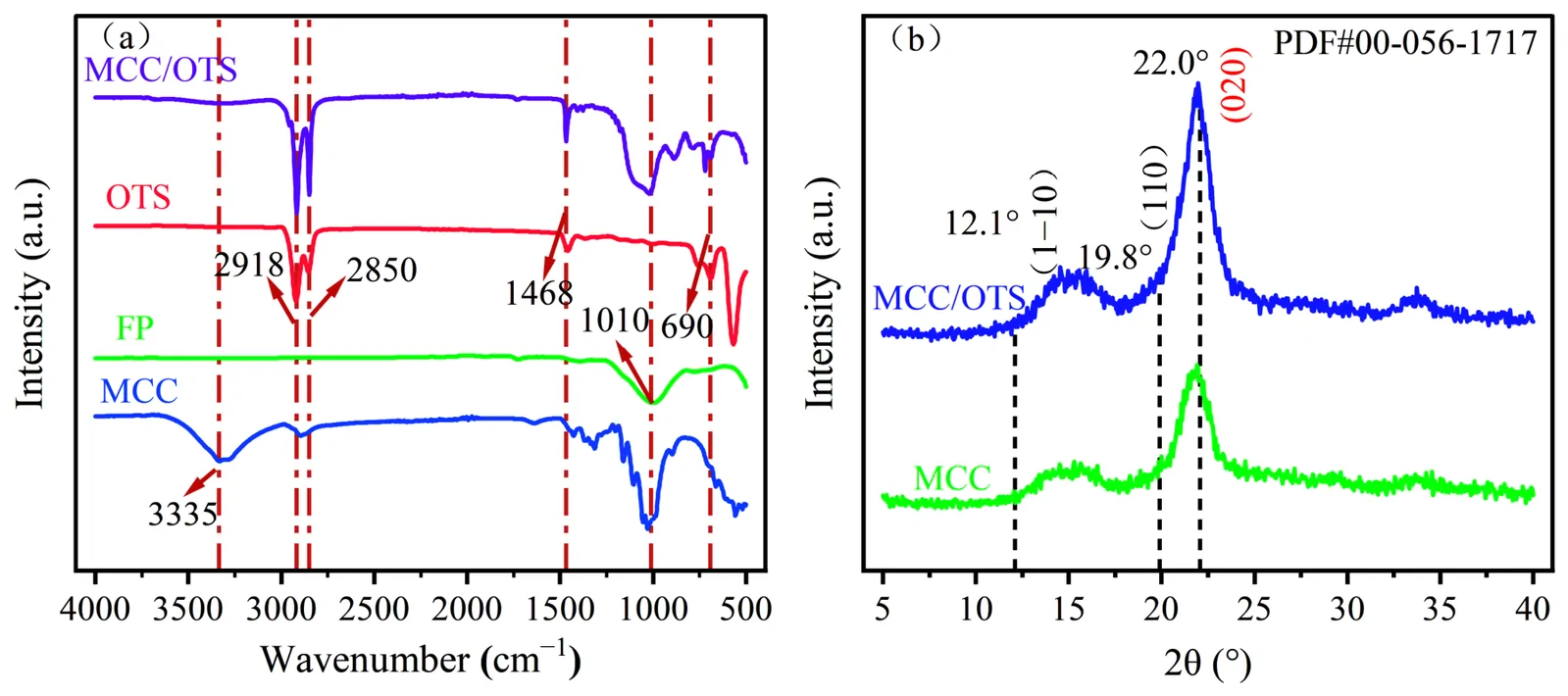
Structural characterization of MCC, FP, OTS and MCC/OTS samples: (**a**) FT-IR spectra of MCC, FP, OTS and MCC/OTS; (**b**) XRD patterns of pristine MCC and MCC/OTS with cellulose II standard card PDF#00-056-1717.

To confirm the chemical structures of the various components and the effectiveness of the modification, Fourier transform infrared (FTIR) spectroscopy was performed on the raw material OTS, pure MCC, cellulose filter paper (FP), and the modified product MCC/OTS. The results are shown in Figure 1a.

In the infrared spectrum of pure MCC, a broad and intense absorption peak appears at 3335 cm^−1^, which is attributed to the -OH stretching vibration in intermolecular and intramolecular hydrogen bonds within the cellulose molecules, indicating that its surface is rich in hydroxyl groups and exhibits hydrophilic properties. In addition, MCC exhibits a peak near 1050 cm^−1^ corresponding to the skeletal vibration of the C-O-C furanose ring, and a peak at 1160 cm^−1^ corresponding to the asymmetric stretching vibration of the C-O-C bridge bond; these are characteristic absorption features of cellulose.

The infrared spectra of cellulose filter paper (FP) exhibit absorption features highly consistent with those of MCC. A broad and intense -OH stretching peak is also present at 3335 cm^−1^, indicating that the FP surface is rich in active hydroxyl groups, which provide ample reactive sites for subsequent functionalization. The weak absorption peak observed near 2898 cm^−1^ is attributed to the C-H stretching vibration in the cellulose molecule. At the same time, the multiple absorption peaks of FP at 1163 cm^−1^, 1107 cm^−1^, and 1055 cm^−1^ correspond, respectively, to the antisymmetric stretching vibration of the C-O-C bond in cellulose glycosidic bonds, the stretching vibration of the C-O bond, and the skeletal vibration of the furanose ring, which fully align with the characteristic cellulose absorption peaks of MCC. It is worth noting that a faint absorption peak appears near 1640 cm^−1^ in the FP spectrum, which may originate from a small amount of bound water physically adsorbed onto the fiber surface during the filter paper preparation process. Furthermore, no distinct characteristic peaks of lignin (such as the aromatic ring vibration at 1595 cm^−1^) or hemicellulose were detected in the FP infrared spectrum, indicating that the filter paper used is of high purity and has a chemical composition essentially identical to that of MCC. The structural homology between FP and MCC confirms that both are typical cellulose materials, providing ideal chemical compatibility and abundant hydrogen-bonding anchor points for the subsequent uniform coating of PDA on the FP surface and the effective loading of MCC/OTS particles.

In the OTS spectrum, two strong absorption peaks appear at 2920 cm^−1^ and 2850 cm^−1^, corresponding to the asymmetric and symmetric C-H stretching vibrations of the methylene group in the long-chain alkyl group, respectively, while the peak near 1465 cm^−1^ corresponds to the C-H bending vibration, and the peak near 690 cm^−1^ is attributed to the stretching vibration of the Si-C bond, confirming the presence of silanol groups and alkyl chains in the OTS molecule.

The infrared spectra of the OTS-hydrophobically modified MCC/OTS samples show significant changes. First, the -OH stretching peak of the original MCC at 3335 cm^−1^ almost disappears, indicating substantial consumption of surface hydroxyl groups of MCC during silylation reaction with OTS, with possible formation of siloxane linkages. At the same time, new absorption peaks appear in the MCC/OTS spectrum: methylene C-H stretching peaks at 2918 cm^−1^ and 2850 cm^−1^, as well as a C-H bending peak at 1467 cm^−1^. These peaks correspond to the characteristic peak positions of the alkyl chains in OTS, confirming that the long-chain alkyl groups of OTS were successfully grafted onto the MCC surface. Furthermore, a Si-C vibrational peak appears near 690 cm^−1^ in the MCC/OTS spectrum, demonstrating successful incorporation of OTS-derived silane moieties; Si-O-C and Si-O-Si linkages may both be formed during hydrolysis-condensation. It is worth noting that the absorption peak of MCC/OTS near 1010 cm^−1^ remains intact; this region likely overlaps the vibrations of the cellulose C-O-C bond and the Si-O-Si bond formed after modification, resulting in an imperceptible shift in the peak position [24].

In summary, the FT-IR results clearly indicate that FP and MCC share the same cellulose chemical backbone and abundant hydroxyl active sites, laying the chemical foundation for subsequent membrane substrate construction and particle loading; meanwhile, alkyl chains originating from OTS are anchored onto the hydroxyl-rich MCC surface via siloxane-related interactions, transforming the MCC surface from hydrophilic to hydrophobic. This infrared analysis provides direct chemical structural evidence for the successful preparation of superhydrophobic cellulose membranes, while also explaining the intrinsic mechanisms by which PDA can firmly adhere to the FP surface (via hydrogen bonding and catechol chemistry) and MCC/OTS particles can be stably sprayed onto the FP/PDA surface.

To assess the impact of OTS hydrophobic alteration on the crystalline architecture of MCC, X-ray diffraction analyzes were conducted on both pure MCC and MCC/OTS, with the findings illustrated in Figure 1b. Both specimens displayed three unique diffraction peaks at 2θ = 12.1°, 19.8°, and 22.0°, which align with the (1−10), (110), and (020) crystal planes of cellulose Type II, respectively (see standard card PDF#00-056-1717). Following the OTS change, there were no notable alterations in the locations or forms of any diffraction peaks, nor did any additional peaks emerge. This suggests that the OTS grafting reaction predominantly took place on the hydroxyl groups of the cellulose surface, preserving the integrity of the crystalline areas within the material, which remained unaltered. Significantly, the diffraction peak strength at 22.0° for MCC/OTS was marginally greater than that of pure MCC, and the peak exhibited a sharper profile, suggesting a little enhancement in crystallinity. This occurrence can be ascribed to two elements: (i) the extended alkyl chains of OTS might have instigated a localized ordered configuration of molecular chains on the cellulose surface during grafting, resulting in a measure of surface orientation; (ii) the application of heat and solvent treatment throughout the modification phase may have facilitated the recrystallization of cellulose’s amorphous regions, consequently enhancing the overall crystallinity. The aforementioned findings validate that OTS modification bestows hydrophobic properties to MCC without compromising its crystalline structure; instead, it somewhat improves the structural uniformity, hence reinforcing the mechanical integrity and interfacial stability of the resultant membrane material [23,25].

**Figure 2:**
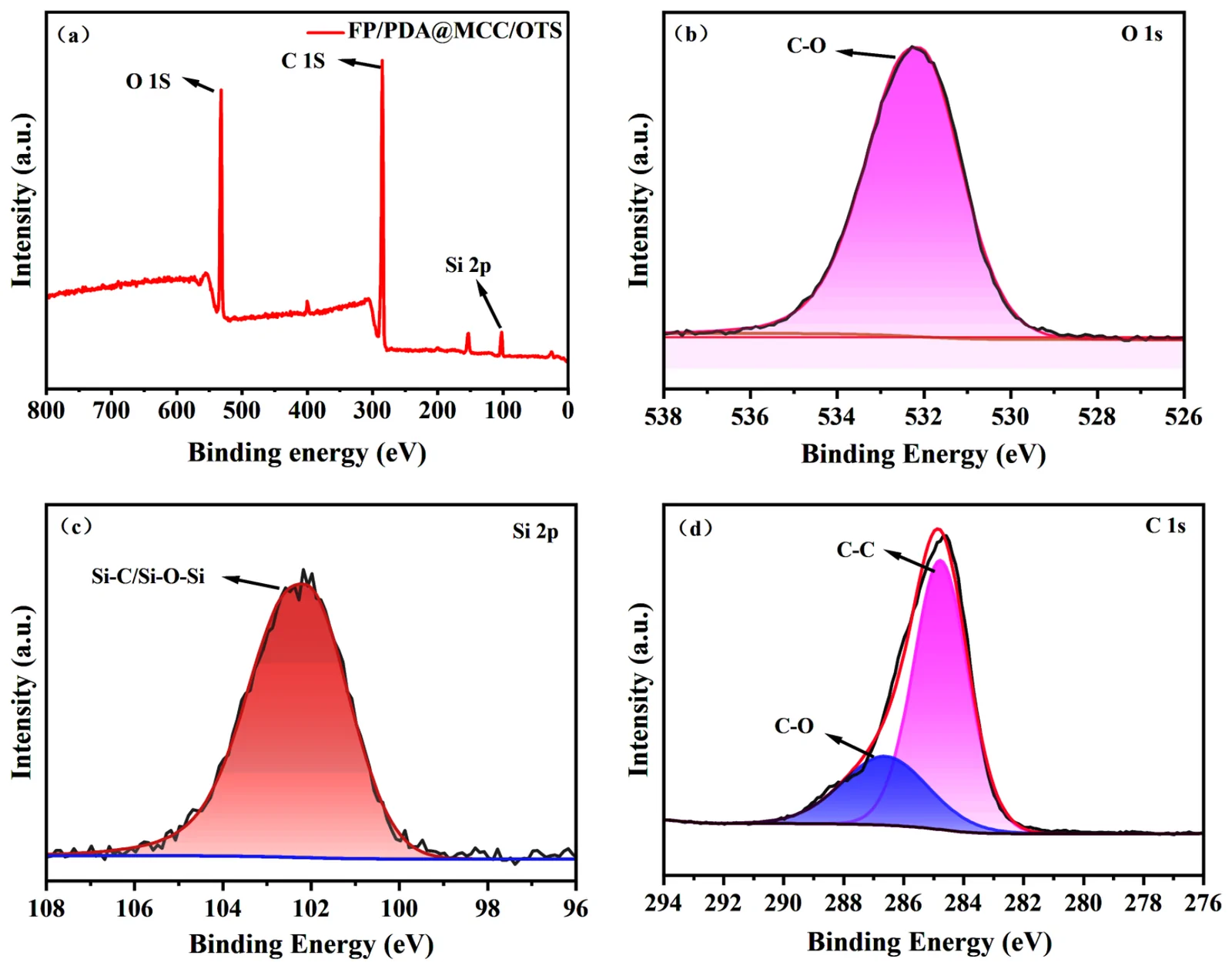
XPS surface chemical analysis of the FP/PDA@MCC/OTS membrane: (**a**) survey spectrum; (**b**) high-resolution O 1s spectrum; (**c**) high-resolution Si 2p spectrum; (**d**) high-resolution C 1s spectrum.

To further verify the successful preparation of the OTS-hydrophobic-modified and PDA-reinforced coating at the surface chemistry level, XPS analysis was performed on the final product FP/PDA@MCC/OTS. The full-spectrum scan results shown in Figure 2a indicate that the sample surface primarily contained three elements: C, O, and Si. A faint N 1s signal (≈400 eV) was also detected, with the N element originating from the amine/imide groups in the PDA layer, while the Si element clearly indicates the introduction of OTS. These full-spectrum results preliminarily confirm that both functional components, PDA and OTS, were successfully loaded onto the surface of the cellulose membrane [23,25].

Further detailed analysis of the high-resolution spectrum (Figure 2d, C 1s) reveals that the two main peaks with binding energies at 284.8 eV and 286.5 eV correspond to C-C (or C-H) bonds and C-O bonds, respectively. The C-O bonds primarily originate from the phenolic hydroxyl groups in the cellulose backbone and PDA, while the C-C bonds originate from the long-chain alkyl groups of OTS and the aromatic ring structures of PDA. Additionally, a shoulder peak at approximately 285.8 eV, attributed to C-N bonds, further confirms the incorporation of PDA.

The high-resolution O 1s spectrum (Figure 2b) exhibits a single asymmetric main peak at 532.2 eV, which can be attributed to the superimposed contributions of C-O bonds (hydroxyl/ether bonds in cellulose and PDA) and Si-O bonds (from the siloxane network formed by the hydrolysis and condensation of OTS); however, since their binding energies are close, they are difficult to distinguish directly.

The Si 2p fine-structure spectrum (Figure 2c) exhibits a distinct characteristic peak at 102.2 eV, indicative of Si-containing species including Si-C and siloxane-type Si-O-Si environments. Due to very close binding-energy values, Si-O-C cannot be unambiguously separated from Si-O-Si solely by XPS, which confirms silane is not merely physically adsorbed on the surface. Based on the above XPS results, combined with the simultaneous detection of Si and N in the full spectrum, the coexistence of alkyl carbon and C-N in the C 1s spectrum, and the characteristic organosilicon bonding states in the Si 2p spectrum, it can be fully confirmed that PDA was effectively coated onto the surface of the filter paper fibers, providing a strong interfacial adhesion layer. Simultaneously, hydrophobic long-chain alkyl moieties from OTS were stably anchored onto MCC particles and were successfully deposited onto the membrane surface by spray-coating. This surface chemical composition closely matches the intended design, providing direct chemical structural evidence for the subsequent superhydrophobic performance and durability [26,27].

### 2.2. SEM Analysis

To investigate the morphological evolution and structural features of the cellulose-based materials at each fabrication stage, scanning electron microscopy (SEM) was performed on pristine filter paper (FP), PDA-toughened filter paper (FP/PDA), pristine microcrystalline cellulose (MCC), OTS-modified MCC (MCC/OTS), and the final superhydrophobic membrane (FP/PDA@MCC/OTS). The corresponding macroscopic photographs are also presented for intuitive comparison.

**Figure 3:**
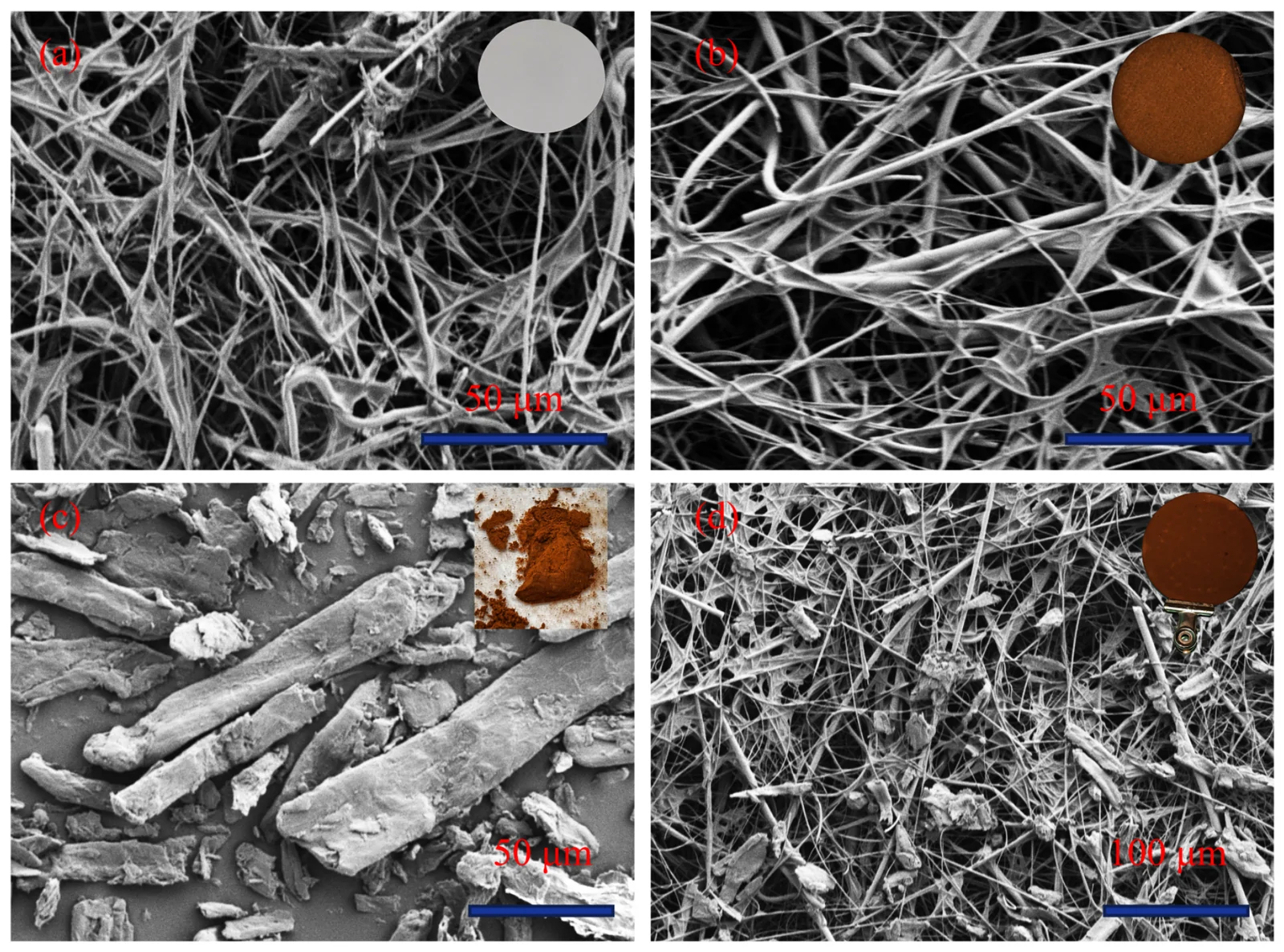
Morphological characterization of samples at different fabrication stages: (**a**) pristine FP; (**b**) FP/PDA; (**c**) MCC particles; (**d**) FP/PDA@MCC/OTS membrane; insets display digital photographs of each specimen.

As shown in Figure 3, pristine FP exhibits a typical three-dimensional intertwined fibrous network with smooth fiber surfaces and numerous inter-fiber pores. Macroscopically, it appears as a white, round filter paper with a uniform texture. After PDA modification (FP/PDA), the microscopic fiber skeleton remains well preserved, yet the fiber surfaces become noticeably rougher and coated with a thin adherent layer, indicating successful deposition of polydopamine [20,27]. Correspondingly, the macroscopic color of FP/PDA changes from white to light brown, further confirming the uniform PDA coating [28].

For the MCC particles, the pristine material shows an irregular granular morphology with relatively smooth surfaces and clear edges, appearing as a white powder macroscopically. In contrast, the OTS-modified MCC/OTS particles retain a similar granular shape but exhibit slightly smoother surface textures and fewer interparticle voids at the microscopic level. This is likely due to the silane grafting, which reduces surface polarity and induces particle aggregation [26]. Macroscopically, MCC/OTS turns from white to brownish-yellow, consistent with the introduction of the hydrophobic alkylsilane layer.

Finally, after spray-coating MCC/OTS onto the FP/PDA substrate, the resulting FP/PDA@MCC/OTS membrane displays a distinctive “fiber-particle” composite structure in the SEM images [29,30]. The cellulose fiber scaffold of the filter paper is still clearly visible, but a large number of MCC/OTS particles are densely and uniformly adhered to the fiber surfaces and inter-fiber junctions, creating a hierarchical rough topography [31]. This rough structure is essential for achieving superhydrophobicity. Macroscopically, the membrane exhibits an overall brownish color with a slightly matte surface, reflecting the successful integration of the hydrophobic particulate coating onto the PDA-toughened fibrous network.

Collectively, the SEM and macroscopic observations demonstrate that PDA modification effectively roughens and functionalizes the filter paper fibers, providing strong interfacial adhesion for subsequent particle deposition [27,28]. Meanwhile, the MCC/OTS particles are successfully loaded without destroying the underlying fibrous framework. This hierarchical architecture, combining microscale fibers and submicron/nanoscale particles, is expected to synergistically enhance surface roughness and facilitate the superhydrophobic wetting state [29,30].

### 2.3. Thermal Stability and Mechanical Properties

**Figure 4:**
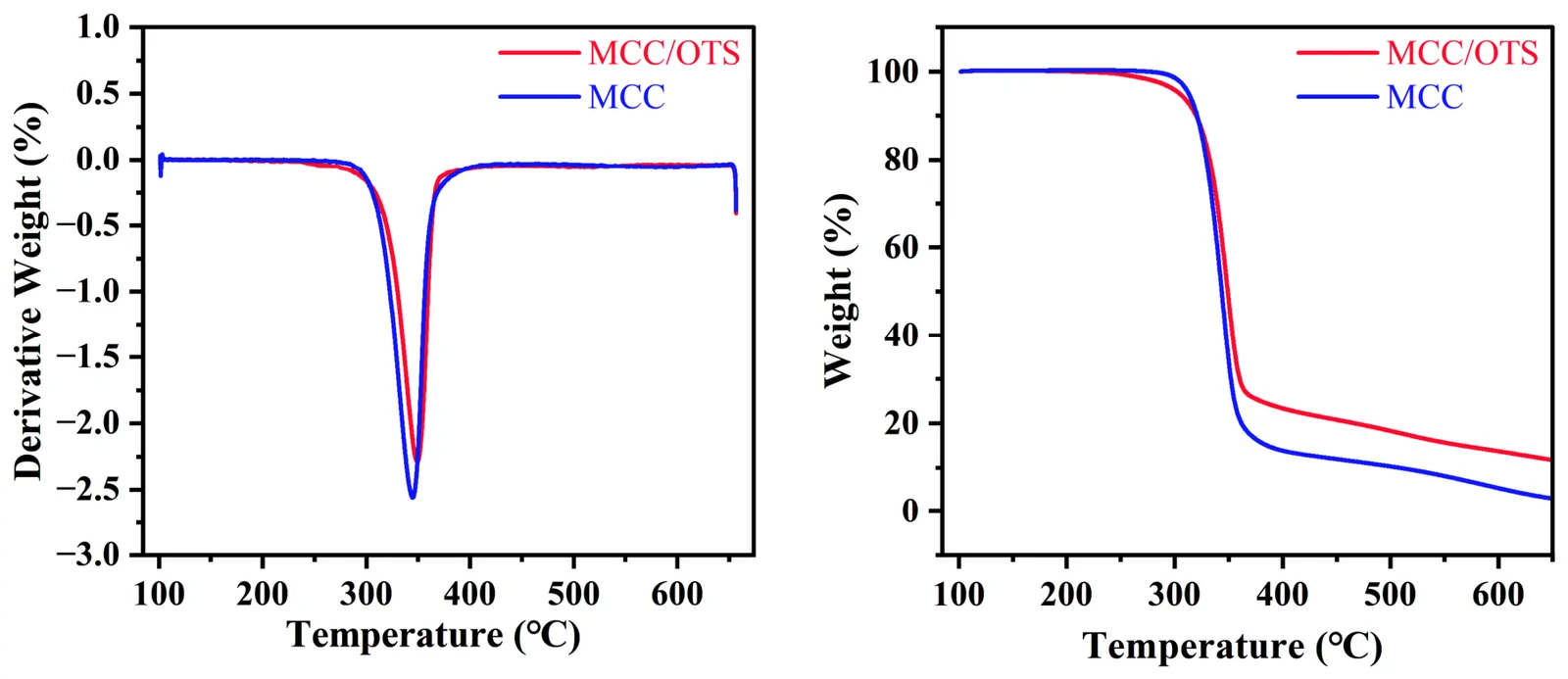
Thermogravimetric analysis of pristine MCC and modified MCC/OTS samples: DTG and TG curves.

Thermogravimetric analysis (TGA) was conducted to evaluate the thermal stability of vacuum-dried pristine MCC and OTS-grafted MCC/OTS, and the corresponding TG and DTG curves are shown in Figure 4. Both samples exhibit a typical three-stage weight-loss profile. The first stage, occurring below ≈150 °C, corresponds to the evaporation of physically adsorbed water and residual solvents [32]. The second stage, between approximately 250 °C and 380 °C, is attributed to the thermal decomposition of cellulose backbone, involving depolymerization, dehydration, and cleavage of glycosidic bonds [33,34]. The final stage above 380 °C involves further carbonization of the residual char.

Notably, the onset decomposition temperature of MCC/OTS (≈250 °C) is slightly lower than that of pristine MCC (≈270 °C). This slight decrease is likely due to the early cleavage of surface-grafted alkylsilane chains or the catalytic effect of residual silanol groups [34]. However, the subsequent degradation of MCC/OTS proceeds more slowly, and its residual mass at 500 °C reaches 14.46%, significantly higher than that of pristine MCC (10.21%). This improved char yield indicates that the OTS grafting layer acts as a protective barrier, retarding the diffusion of volatile degradation products and promoting cross-linking reactions that enhance thermal resistance [26,35].

The derivative thermogravimetric (DTG) curves further support this conclusion. The maximum decomposition temperature (T_max_) of pristine MCC appears at 340–350 °C, while that of MCC/OTS shifts to approximately 360 °C, with a notably shallower peak. The upward shift in T_max_ and the reduced mass-loss rate confirm that OTS modification effectively delays the thermal degradation process [24]. This enhanced thermal stability ensures that the superhydrophobic membrane can withstand the typical temperature variations encountered in oily wastewater treatment scenarios (e.g., hot oil-water mixtures or solar-heated systems) without significant performance degradation [14].

In addition to thermal stability, the mechanical properties of the resulting membrane are crucial for practical applications. The PDA interfacial layer not only improves adhesion but also contributes to the overall mechanical robustness of the composite. The FP/PDA@MCC/OTS membrane retained its superhydrophobicity after repeated wear cycles in abrasion tests (Section 2.4), indicating that the PDA-reinforced structure effectively mitigates coating detachment under mechanical stress. This synergistic effect of thermal and mechanical durability is consistent with previous reports on polydopamine-enhanced cellulose composites [27,28].

### 2.4. Surface Wettability, Abrasion Resistance, Acid-Base Stability, and Durability Test

**Figure 5:**
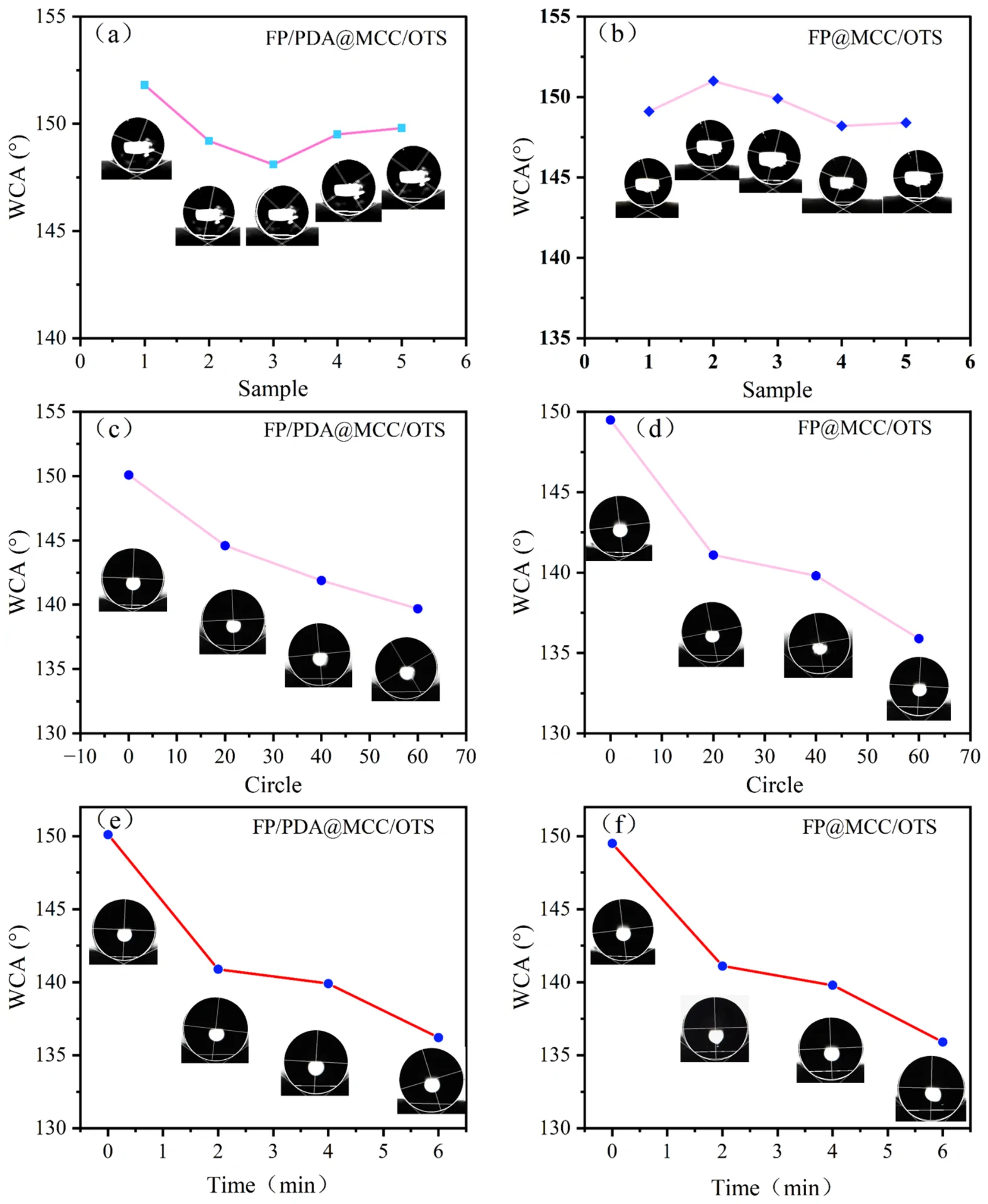
Water-repellent performance of FP/PDA@MCC/OTS and FP@MCC/OTS membranes: (**a**,**b**) batch-to-batch repeatability of as-prepared membranes; (**c**,**d**) water contact angle variation after different steel-wool abrasion cycles; (**e**,**f**) water contact angle evolution under ultrasonic treatment in anhydrous ethanol; insets show optical photographs of water droplets on the membrane surface.

Surface wettability was first evaluated by water contact angle (WCA) measurements. The real-time WCA measurement process on the membrane surface is presented in Supplementary Video S3. As shown in (Figure 5a,b), the PDA-reinforced membrane (FP/PDA@MCC/OTS) exhibited an average WCA of 150.1° (maximum of 151.8°), slightly higher than that of the unreinforced counterpart FP@MCC/OTS (average of 149.5°, maximum of 151°). Both values exceed the superhydrophobic threshold of 150°, confirming the successful grafting of OTS [36]. The higher WCA of the PDA-modified sample indicates that the PDA interlayer enhances the adhesion and uniform deposition of hydrophobic particles, resulting in increased surface coverage. This enhanced adhesion is attributed to the catechol and amine groups of PDA, which establish robust hydrogen-bonding and catechol-mediated interfacial interactions with cellulose substrates [37,38]. The dynamic water repellency of the membrane was further verified by droplet impact tests, in which water droplets bounced off the membrane surface upon impact (Supplementary Video S1) and slid away rapidly without pinning on the tilted membrane (Supplementary Video S6). Moreover, the sliding angle (SA) of the membrane was measured to be ≈5° (Supplementary Video S2), confirming ultra-low droplet adhesion.

Abrasion resistance was assessed under a constant 20 g load using steel wool as the abrasive medium (Figure 5c,d). For FP/PDA@MCC/OTS, the WCA decreased from 150.1° initially to 144.6° after 20 cycles, 141.9° after 40 cycles, and 136.2° after 60 cycles. In contrast, FP@MCC/OTS dropped to 141.1°, 139.8°, and 135.9° under the same conditions. Although both membranes suffered gradual WCA loss due to mechanical removal of hydrophobic particles, the PDA-toughened sample consistently retained higher WCAs, demonstrating that PDA effectively reinforces particle–substrate adhesion and mitigates coating detachment [37,38]. The complete 60-cycle abrasion process under a 20 g load and the post-abrasion WCA measurements on the worn areas are documented in Supplementary Video S4.

Durability under ultrasonic treatment (300 W in anhydrous ethanol) is presented in Figure 5e,f. After 2, 4, and 6 min of sonication, FP/PDA@MCC/OTS maintained WCAs of 140.0°, 139.9°, and 136.2°, respectively, whereas FP@MCC/OTS showed 138.1°, 136.6°, and 131.3°. The better retention of superhydrophobicity of the PDA-reinforced membrane again underscores the protective role of the polydopamine layer against particle peeling induced by ultrasonic cavitation [39].

**Figure 6:**
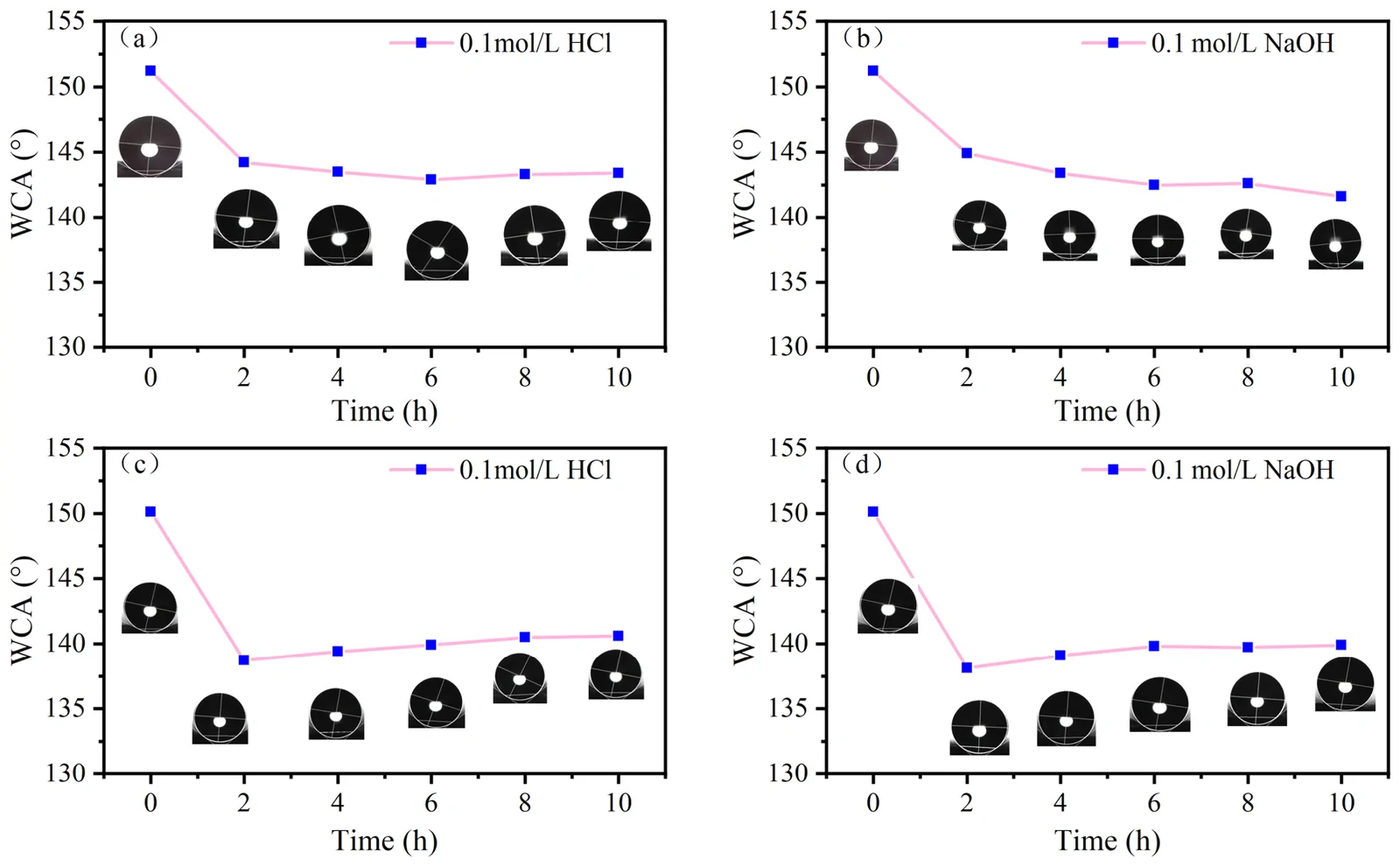
Chemical stability evaluation of FP/PDA@MCC/OTS and FP@MCC/OTS membranes: (**a**,**b**) water contact angle curves of FP/PDA@MCC/OTS immersed in 0.1 mol/L HCl and 0.1 mol/L NaOH; (**c**,**d**) water contact angle curves of FP@MCC/OTS immersed in 0.1 mol/L HCl and 0.1 mol/L NaOH; insets show optical photographs of water droplets on the membrane surface.

Chemical stability was evaluated by immersing both membranes in 0.1 mol/L HCl and 0.1 mol/L NaOH aqueous solutions for up to 10 h (Figure 6). For FP/PDA@MCC/OTS, the WCA remained above 143° in HCl and above 141° in NaOH throughout the entire immersion period (e.g., 144.2° and 144.9° at 2 h, 143.4° and 141.6° at 10 h for HCl and NaOH, respectively). In contrast, FP@MCC/OTS showed WCAs of 138–140° in both acidic and alkaline media, clearly lower than those of the PDA-reinforced sample. These results confirm that the PDA-toughened membrane exhibits outstanding corrosion resistance, with WCA values staying above 140° even after prolonged exposure to harsh chemical environments [39,40].

Collectively, the wettability, abrasion, ultrasonic, and corrosion tests consistently demonstrate that the PDA interfacial layer not only preserves the superhydrophobic state but also significantly enhances mechanical robustness and chemical durability. The FP/PDA@MCC/OTS membrane therefore exhibits better overall stability than the non-reinforced control, validating the effectiveness of the PDA-assisted design for practical oil-water separation under demanding conditions [41,42].

### 2.5. Oil-Water Separation Performance

**Figure 7:**
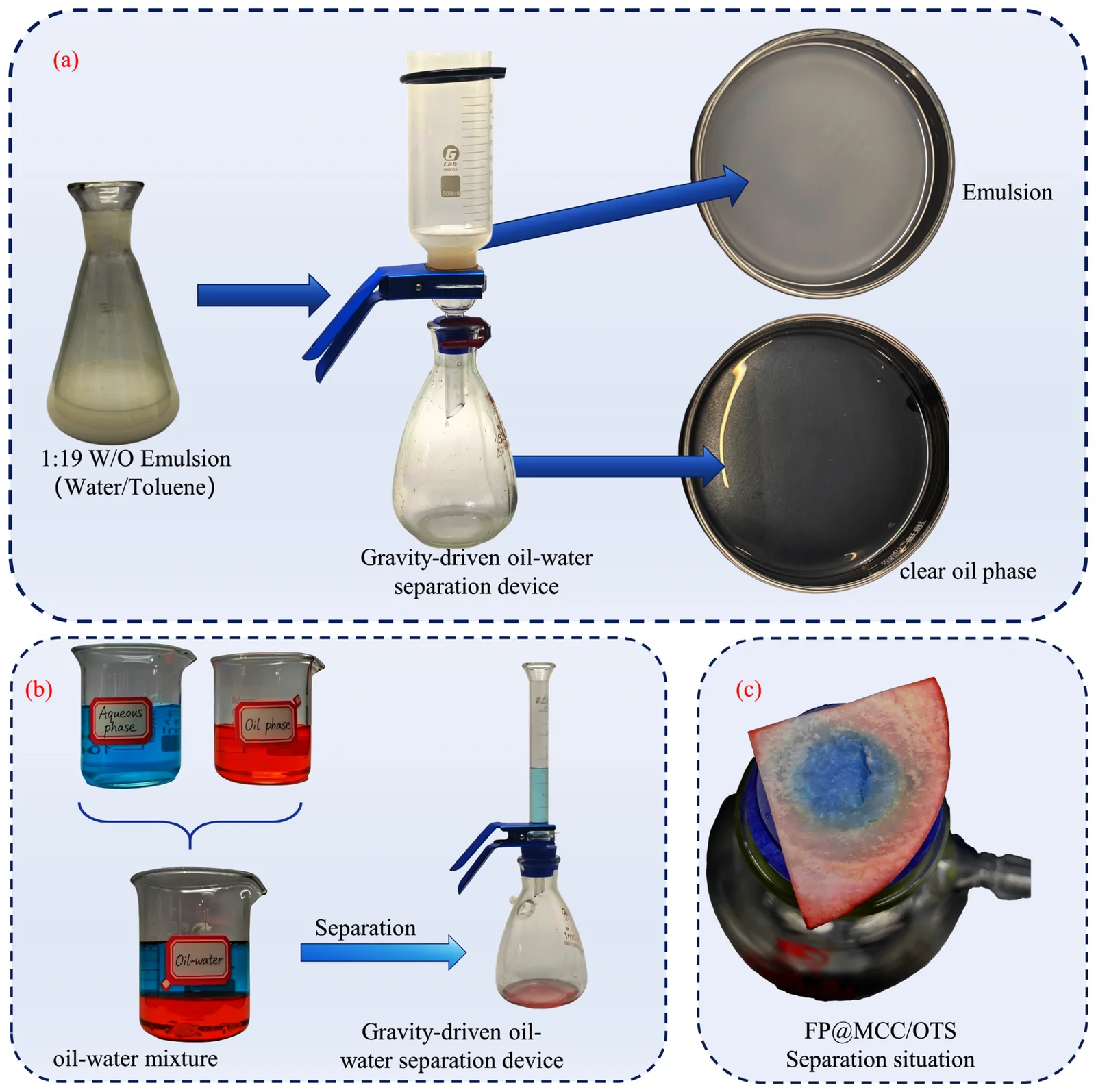
Oil-water separation performance of the as-prepared membranes: (**a**) schematic of surfactant-stabilized water-in-oil emulsion separation; (**b**) schematic of gravity-driven oil-water mixture separation; (**c**) separation failure of unreinforced FP@MCC/OTS membrane.

The oil-water separation performance of the prepared membranes was systematically evaluated using a gravity-driven filtration apparatus. As illustrated in Figure 7c and demonstrated in Supplementary Video S7, the device comprises an upper glass tube for feedstock loading and a lower collection flask, with the membrane sample firmly clamped between them. When a heavy oil/water mixture—composed of 1,2-dichloroethane or tetrachloroethylene (stained with Sudan III) and deionized water (stained with methylene blue) in a 1:1 volume ratio—was poured into the upper tube, the oil phase rapidly permeated through the membrane under gravity, while the water phase was completely retained above the membrane surface. This selective permeation behavior arises from the synergistic effect of the hierarchical fiber-particle roughness and the low-surface-energy alkylsilane grafting, which together create a superhydrophobic/superoleophilic wetting state consistent with the classical Cassie-Baxter model [43].

**Figure 8:**
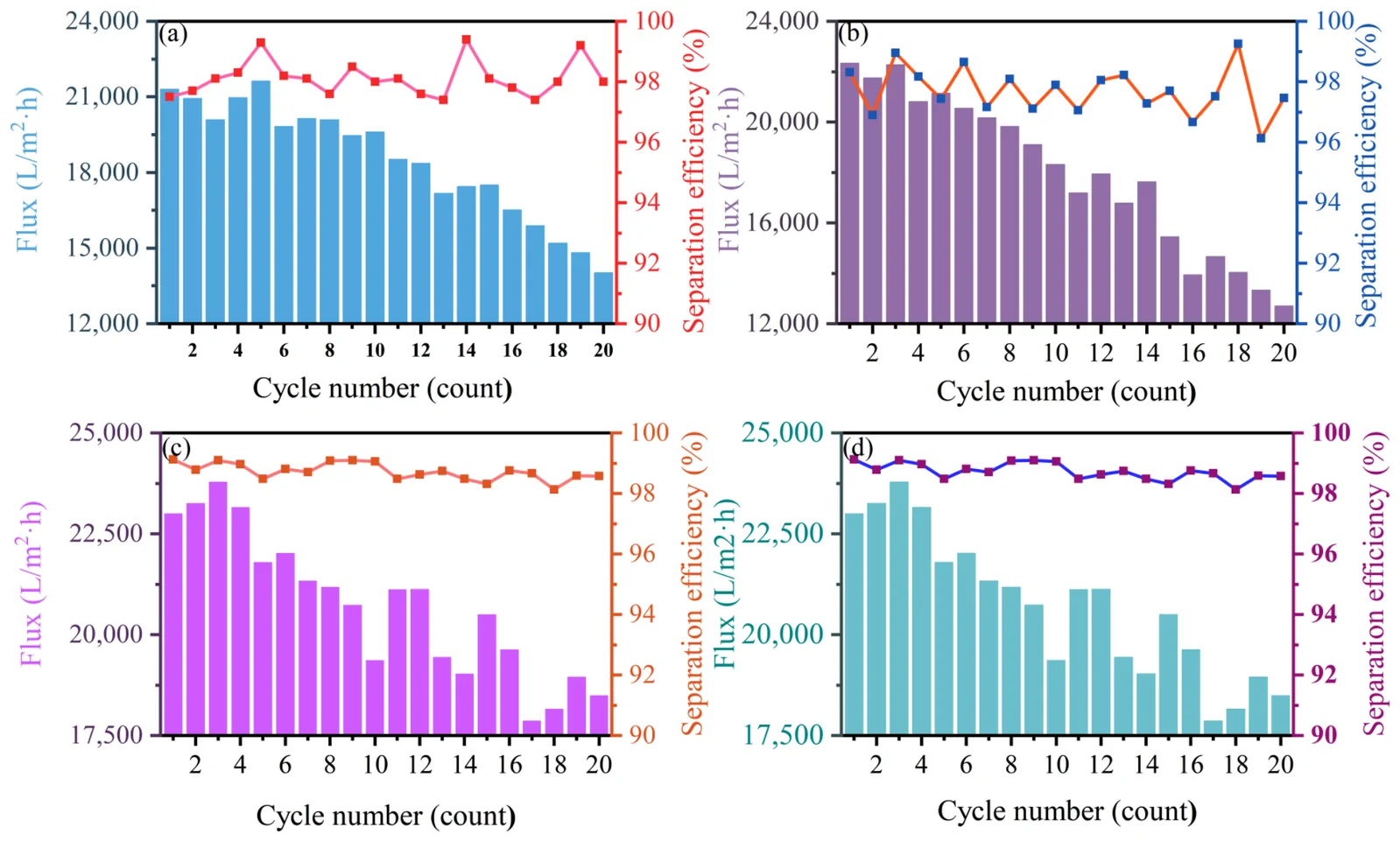
Cyclic separation performance of the prepared membranes: (**a**) FP/PDA@MCC/OTS with 1,2-dichloroethane; (**b**) FP@MCC/OTS with 1,2-dichloroethane; (**c**) FP/PDA@MCC/OTS with tetrachloroethylene; (**d**) FP@MCC/OTS with tetrachloroethylene.

Figure 8 presents the separation efficiency and flux of both FP/PDA@MCC/OTS and FP@MCC/OTS over 20 cyclic separation runs (detailed data for each cycle are summarized in Supplementary Table S1). For the PDA-reinforced FP/PDA@MCC/OTS membrane, the separation efficiency remained consistently above 98% throughout all cycles, reaching a maximum of 99.99% for the 1,2-dichloroethane/water system and 99.12% for the tetrachloroethylene/water system. The corresponding separation fluxes were as high as 21,303.83 L·m^−2^·h^−1^ and 22,997.26 L·m^−2^·h^−1^, respectively. These values are comparable to or even higher than those of recently reported superhydrophobic cellulosic membranes prepared by similar silanization strategies [19,23], and they approach the theoretical upper bound for gravity-driven permeation through macroporous fibrous media. In contrast, the non-reinforced FP@MCC/OTS membrane exhibited slightly lower performance, with maximum separation efficiencies of 99.26% and 99.10%, and maximum fluxes of 20,948.91 L·m^−2^·h^−1^ and 17,238.77 L·m^−2^·h^−1^, respectively. The superior and more stable performance of FP/PDA@MCC/OTS is attributed to the polydopamine interfacial layer. Through strong hydrogen-bonding and catechol-mediated adhesion [44], this layer enhances the uniform deposition and anchoring of hydrophobic MCC/OTS particles onto the cellulose fiber skeleton [45], thereby preserving the hierarchical surface topography and superhydrophobic state during repeated operation.

To contextualize the performance of our FP/PDA@MCC/OTS membrane, we compared its key metrics with representative 2016–2026 studies on OTS- or PDA/OTS-modified cellulose-based oil-water separation membranes. Wang et al. (2016) [46] obtained superhydrophobic filter paper with ≈99.4% separation efficiency, but no flux or durability data were provided. Yang et al. (2021) [47] prepared PDA + OTS-coated O-PASS films with a low WCA of 132.6° and a small flux of 10–34 L·m^−2^·h^−1^ for water-in-oil emulsions. Ning et al. (2025) [23] reported SiO_2_ + OTS dip-coated cellulose paper (WCA = 157°, efficiency = 99.31%, flux ≈ 5.1 × 10^3^ L·m^−2^·h^−1^), yet its coating degraded after 50 abrasion cycles under 5–10 g load. The recent multi-step dip-coated PDA@BN@SiO_2_@OTS (2026) [48] reached a high WCA (160–165°) and tolerated 40 cycles at ≈10 g, though flux and acid-base stability were not given. In comparison, our membrane delivers a flux of ≈2.2 × 10^4^ L·m^−2^·h^−1^ (four times higher than Ning’s work). It survives 60 abrasion cycles under 20 g load with a WCA above 136°, and maintains a WCA > 140° after 10 h immersion in solutions with a pH of 1–14. These comparisons demonstrate that our PDA-reinforced spray-coating strategy achieves a favorable combination of high flux, good mechanical durability and chemical resistance, which has not been simultaneously achieved in previous OTS-based cellulose membranes. The detailed comparison is summarized in Supplementary Table S2 [23,46,47,48].

Figure 7c provides a photographic comparison of the separation process using FP@MCC/OTS. It is clearly observed that, although the non-reinforced membrane initially allowed oil passage while blocking water, it became prone to structural rupture and cracking under the continuous gravitational stress, particularly after multiple separation cycles. This mechanical failure resulted in direct leakage of the water phase into the collection flask, leading to a pronounced decline in separation efficiency and poor reusability. Such brittle behavior is a common shortcoming of conventional particle-coated cellulose papers lacking a robust interfacial binder. In sharp contrast, the FP/PDA@MCC/OTS membrane maintained its structural integrity throughout the cyclic tests without any visible damage, underscoring the critical role of the PDA toughening layer in mitigating particle detachment and reinforcing the inter-fiber junctions. This enhanced mechanical robustness is consistent with previous findings on polydopamine-enhanced superhydrophobic coatings, where the adhesive interphase effectively improves abrasion and peel resistance [39].

Beyond free oil/water mixtures, the membrane also demonstrated promising performance in separating surfactant-stabilized oil-in-water emulsions, as shown in Figure 7a. The filtrate collected after emulsion separation appeared optically clear, indicating effective coalescence and removal of micron-sized oil droplets. This demulsification capability is attributed to the preferential wetting and capture of emulsified oil droplets by the superhydrophobic particle layer, which promotes droplet coalescence and subsequent permeation through the macroporous cellulose network.

Collectively, the oil-water separation results demonstrate that the PDA-assisted interfacial engineering strategy not only preserves the superhydrophobicity and high permeation flux of the cellulose membrane but also significantly enhances its mechanical durability and anti-rupture resistance during dynamic filtration operations. This robust performance, combined with the scalable spray-coating fabrication process, renders the FP/PDA@MCC/OTS membrane a highly promising candidate for practical treatment of oily wastewater under demanding industrial conditions.

## 3. Materials and Methods

### 3.1. Materials

Micron-sized cellulose powder (particle size of 8–10 μm, degree of polymerization ≈ 220, crystallinity index ≈ 75%, sourced from Shanghai McLean Biochemical Technology Co., Ltd., Shanghai, China, henceforth referred to as McLean), triethylamine (analytical grade, McLean), octadecyltrichlorosilane (purity > 95%, McLean), dopamine hydrochloride (reagent grade, McLean), glacial acetic acid (>95%, McLean), n-hexane (analytical grade, McLean), toluene (analytical grade, McLean), and anhydrous ethanol (analytical grade, McLean) were used. Glass-coated hydrophilic cellulose filter paper (diameter 90 mm, pore size 1.2 μm) was supplied by Haining Delv New Material Technology Co., Ltd., Haining, China. All ingredients and reagents were used exactly as supplied, requiring no additional purification.

### 3.2. Preparation Process of Oil-Water Separation Membranes

**Figure 9:**
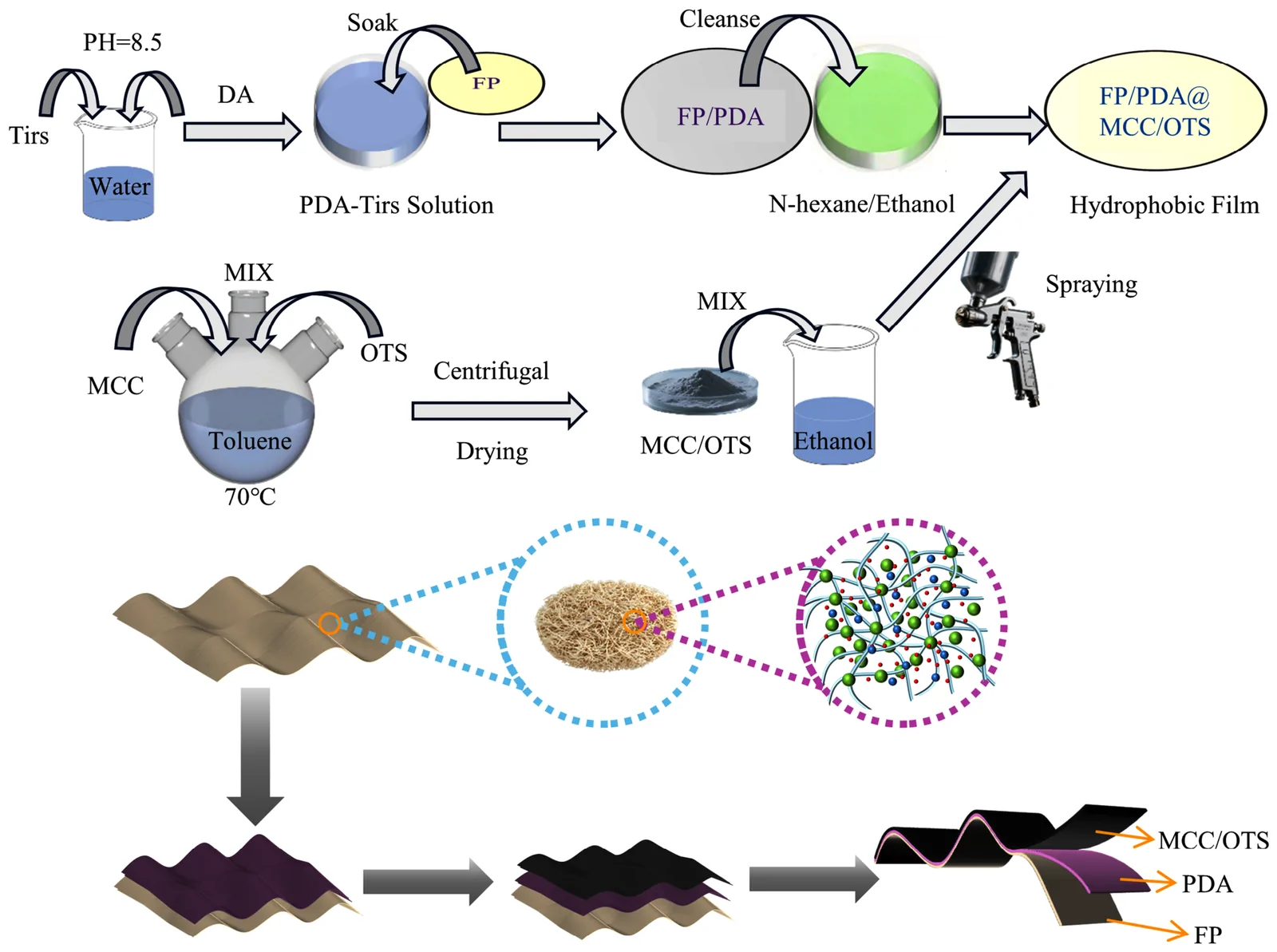
Schematic illustration for the preparation and hierarchical structure of FP/PDA@MCC/OTS composite membrane.

As illustrated in Figure 9, first, a 2% dopamine hydrochloride-tris (hydroxymethyl)aminomethane (Tris) buffer solution (pH 8.5) was prepared by adding 1 g of dopamine hydrochloride to a 1 mol/L Tris solution that had been adjusted to pH 8.5 with 1 mol/L hydrochloric acid. Cellulose filter paper (FP) was then submerged in this solution for 30 min, dried at 80 °C and 60% humidity for 12 h, and further dried at 80 °C for 3 h. To obtain the toughened filter paper (FP/PDA), the paper was saturated with n-hexane and anhydrous ethanol for 30 min each and then dried at 80 °C for 3 h. Separately, 2 g of microcrystalline cellulose (MCC) were dispersed in toluene after being vacuum-dried for 6 h. Triethylamine was used as a catalyst in a 12 h reflux reaction with octadecyltrichlorosilane (OTS) in a 70 °C oil bath. The mixture was centrifuged, neutralized with methanol, and vacuum-dried for 6 h to obtain hydrophobic-modified cellulose (MCC/OTS). The spray suspension was prepared by dispersing MCC/OTS in anhydrous ethanol, and then applied to the FP/PDA surface in three consecutive spraying steps. After each spray, the sample was temporarily dried at 80 °C, and finally dried at 80 °C and 60% humidity for 12 h. After washing with n-hexane and anhydrous ethanol for 10 min, the sample was dried at 80 °C for 3 h to yield the superhydrophobic cellulose membrane (FP/PDA@MCC/OTS). For comparison, the control sample FP@MCC/OTS was prepared by directly spraying the MCC/OTS suspension onto the surface of unmodified FP using the same procedure.

### 3.3. The Study on Oil and Water Separation

The procedure outlined in Reference [23] was used to generate a heavy oil/water mixture: 1,2-dichloroethane (or tetrachloroethylene) and water were combined in a 1:1 volume ratio (20 mL each); Sudan III was used to stain the oil phase, and methylene blue was used to stain the aqueous phase. The mixture was placed into the top glass tube for oil-water separation after the manufactured superhydrophobic cellulose membranes (FP/PDA@MCC/OTS or FP@MCC/OTS) were installed in a gravity-driven separation apparatus.

For the preparation of water-in-oil (W/O) emulsions, 5 mL of deionized water (aqueous phase) and 100 mL of toluene (light oil phase) were mixed with 0.1 g of Span 80 (sorbitan monooleate) as the surfactant. The mixture was then ultrasonicated at 500 W for 20 min using an ultrasonic oscillator to obtain a stable W/O emulsion. The as-prepared emulsion was subsequently subjected to separation using the same gravity-driven filtration setup to evaluate the demulsification performance of the superhydrophobic membranes.

The following formula was used to determine the separation efficiency (*R*):

(1) $$R=\frac{m_{1}}{m_{2}}\times 100\%$$

where *m*_1_ is the original oil mass, and *m*_2_ is the mass of the collected filtrate [19]. The separation flux (*J*) was computed using this method:

(2) $$J=\frac{V}{S\times \Delta t}$$

where *V* is the volume of the filtrate (L), Δ*t* is time (h), and *S* is the effective membrane area (m^2^) [19,35].

### 3.4. Characterization

Fourier-transform infrared spectroscopy (FTIR, PE-FTIR, PerkinElmer, Waltham, MA, USA, 4000–400 cm^−1^, resolution 4 cm^−1^, 32 scans) in attenuated total reflection mode was used to examine the samples’ chemical structures. X-ray diffraction (XRD, TD-3500, Dandong Tongda Science & Technology Co., Ltd., Dandong, China; tube voltage of 40 kV, tube current of 40 mA, scan range of 5–40°, step size of 0.05°, scan speed of 3°/min) was used to examine crystallinity. A scanning electron microscope (SEM, Apreo 2S, Thermo Fisher Scientific, Waltham, MA, USA) was used to examine the sample morphology. An optical contact angle measuring device was used to measure the water contact angle (WCA) (YK-2000, Shenzhen Yuanke Instrument Equipment Co., Ltd., Shenzhen, China), and the average value was computed from at least three separate points on each sample. A universal testing machine (RK-339-GSR, Shenzhen Runke Instrument Equipment Co., Ltd., Shenzhen, China) was used to test the mechanical properties (tensile rate of 0.5 mm/min, gauge length of 20 mm; at least five independent specimens were examined for each sample at room temperature). A thermogravimetric analyzer (TGA, TGA 4000, PerkinElmer, Waltham, MA, USA) operating in a dynamic nitrogen environment was used to assess thermal stability. The analyzer was heated from 25 °C to 100 °C at a rate of 10 °C per minute, held for 10 min to eliminate adsorbed water, and then heated to 800 °C at a rate of 10 °C per minute. Using a 50 g weight and steel wool as the abrasive medium, the coated film was subjected to 100 back-and-forth abrasion cycles to assess its resistance to abrasion. Following abrasion, morphological changes were noted, and the oil-water separation performance was assessed.

## 4. Conclusions

In summary, we successfully fabricated a durable superhydrophobic cellulose-based membrane (FP/PDA@MCC/OTS) via a straightforward impregnation–spray coating strategy. The key innovation lies in the introduction of a polydopamine interfacial toughening layer, which not only preserves the intrinsic fibrous architecture of the filter paper but also provides robust adhesion for the subsequent spray-deposited OTS-grafted MCC particles. This hierarchical fiber-particle composite structure, combined with low-surface-energy alkylsilane grafting, endows the membrane with stable superhydrophobicity (average WCA > 150°) and excellent oleophilicity, enabling selective oil permeation under gravity-driven conditions.

The resulting membrane demonstrates outstanding oil-water separation performance, with an average separation efficiency exceeding 98% (up to 99.99% for 1,2-dichloroethane/water mixtures) and a high permeation flux of up to 2.3 × 10^4^ L·m^−2^·h^−1^. More importantly, the PDA-reinforced membrane exhibits significantly enhanced durability compared to its non-reinforced counterpart. For example, the WCA consistently remained above 140° after 60 abrasion cycles, 6 min of ultrasonic treatment, or 10 h of immersion in 0.1 mol/L HCl or NaOH solutions. These results unequivocally demonstrate that the PDA interlayer effectively mitigates particle detachment and coating degradation under mechanical and chemical stresses, a critical advantage over conventional direct-silanization approaches [23].

The mechanistic insight gained from this work is twofold. First, the catechol- and amine-rich PDA layer establishes robust hydrogen-bonding and catechol-mediated interfacial interactions with both the cellulose substrate and MCC/OTS particles, creating a cohesive interface that resists delamination. Second, the spray-coating process enables uniform deposition of hydrophobic particles onto the three-dimensional fibrous network without compromising its macroporous structure, thereby maintaining high permeation flux while achieving extreme surface wettability. This synergistic combination of interfacial toughening and hierarchical roughness engineering offers a generalizable platform for upgrading cellulose-based materials toward durable superhydrophobic applications.

Beyond its immediate application in oily wastewater treatment, this work underscores the broader potential of bio-derived polymers—when rationally modified with bioinspired adhesives like PDA—to replace conventional synthetic and fluorinated coatings in sustainable separation technologies. The fabrication process is simple, scalable, and uses commercially available reagents, rendering it readily adaptable for industrial production. We anticipate that this PDA-assisted strategy can be extended to other cellulose-derived substrates (e.g., fabrics, sponges, aerogels) and other functional particles, opening new avenues for designing durable, eco-friendly, and high-performance materials for water remediation and beyond.

## Data Availability

All data supporting the findings of this study are included within the. article and its Supplementary Materials.

## Supplementary Materials

The following supporting information can be downloaded at https://doi.org/10.5281/zenodo.22843017 (accessed on 19 September 2026). Table S1: Complete oil-water separation performance data over 20 cycles; Table S2: Comparison of oil-water separation performance of FP/PDA@MCC/OTS with that of previously reported OTS-based cellulose membranes; Video S1: Water droplet bouncing test on FP/PDA@MCC/OTS membrane; Video S2: Sliding angle measurement (≈5°) on tilted membrane; Video S3: Real-time WCA measurement on membrane surface; Video S4: Full 60-cycle abrasion process (20 g steel wool) and post-abrasion WCA measurement on worn areas; Video S5: Gravity-driven oil-water separation test showing heavy oil permeation; Video S6: Droplet impact and sliding on membrane surface; Video S7: Demonstration of the gravity-driven oil-water separation process.

## Abbreviations

The following abbreviations are used in this manuscript:

FP	Cellulose filter paper
PDA	Polydopamine
MCC	Microcrystalline cellulose
OTS	Octadecyltrichlorosilane
